# A theoretical framework to describe communication processes during medical disability assessment interviews

**DOI:** 10.1186/1471-2458-9-375

**Published:** 2009-10-06

**Authors:** H Jolanda van Rijssen, Antonius JM Schellart, Johannes R Anema, Allard J van der Beek

**Affiliations:** 1 VU University Medical Center, Department of Public and Occupational Health, EMGO Institute for Health and Care Research, Amsterdam, The Netherlands; 2 Research Center for Insurance Medicine, collaboration between AMC-UWV-VUmc, Amsterdam, The Netherlands

## Abstract

**Background:**

Research in different fields of medicine suggests that communication is important in physician-patient encounters and influences satisfaction with these encounters. It is argued that this also applies to the non-curative tasks that physicians perform, such as sickness certification and medical disability assessments. However, there is no conceptualised theoretical framework that can be used to describe intentions with regard to communication behaviour, communication behaviour itself, and satisfaction with communication behaviour in a medical disability assessment context.

**Objective:**

The objective of this paper is to describe the conceptualisation of a model for the communication behaviour of physicians performing medical disability assessments in a social insurance context and of their claimants, in face-to-face encounters during medical disability assessment interviews and the preparation thereof.

**Conceptualisation:**

The behavioural model, based on the Theory of Planned Behaviour (TPB), is conceptualised for the communication behaviour of social insurance physicians and claimants separately, but also combined during the assessment interview. Other important concepts in the model are the evaluation of communication behaviour (satisfaction), intentions, attitudes, skills, and barriers for communication.

**Conclusion:**

The conceptualisation of the TPB-based behavioural model will help to provide insight into the communication behaviour of social insurance physicians and claimants during disability assessment interviews. After empirical testing of the relationships in the model, it can be used in other studies to obtain more insight into communication behaviour in non-curative medicine, and it could help social insurance physicians to adapt their communication behaviour to their task when performing disability assessments.

## Background

In addition to their curative tasks, physicians also often perform different types of medical assessments, such as those that are needed for sickness certification, disability legislation, and social insurance. National standards for these medical assessments vary considerably, but there are several basic principles. In this paper, social insurance medicine in the Netherlands will serve as an example. An important task of physicians working in this field of medicine is to assess the medical status or work capacity of employees with prolonged sick-leave. The medical assessment is the first step in determining whether or not the employee, or claimant, is entitled to social security benefits. In addition to the available information and a physical examination, the key component of this medical assessment is the assessment interview, during which the claimant and the physician meet face-to-face. This interview differs from an ordinary physician-patient encounter, because it is of a less voluntary nature than a physician-patient encounter in curative medicine (i.e. the people who are involved have no choice with regard to participation in the assessment interview) and the physician's assessment has legal consequences for the claimant. The social insurance physician's assessment of the employee's work capacity determines the entitlement to social security benefits [1-3]. The attitude and communication behaviour of the social insurance physician during the assessment is likely to influence the behaviour and cooperation of the claimant, and may thus influence the quality of the information that is obtained and the accuracy of the disability assessment. Similarly, the attitude of the claimant and the claimant's coping behaviour will also influence the content and course of the communication during the assessment, and the quality of the information that the physician receives from the claimant.

### Objective

In social insurance medicine, the style and content of communication behaviour may not only influence the disability assessment process, but possibly also the outcome of the assessment. In view of the influence of communication behaviour in these physician-claimant encounters, and in order to gain insight into the complexity and dynamics of this behaviour, it is important to develop a conceptualised theoretical framework. Therefore, the objective of this article is to describe the conceptualisation of a model for the communication behaviour of social insurance physicians and of their claimants in face-to-face encounters during medical disability assessment interviews. This conceptualisation will be based on the Theory of Planned Behaviour (TPB), and the main relationships in the TPB will be discussed in the context of disability assessment interviews. Along the lines of this theory, we will refer to literature indicating that communication behaviour of social insurance physicians during assessment interviews can be predicted from a combination of their attitudes, experienced social influence, self-efficacy, intentions with regard to behaviour, skills, and barriers for communication with claimants in general. Analogously, we will present literature findings to indicate that the communication behaviour of claimants during the assessment interview can be predicted from their attitudes, intentions, skills, and barriers for their communication with social insurance physicians, or communication with physicians in general if they had no prior experience with social insurance physicians.

### The importance of communication behaviour

Communication is generally defined as a process of transferring information from one source to another. This broad definition is also applicable to the transfer of information between the social insurance physician and the claimant, i.e. the behavioural process of reciprocal contact between social insurance physician and claimant during their face-to-face assessment interview, aimed at (verbal and non-verbal) continuous, dynamic, two-directional information exchange. Information exchange is used here as a broad term to describe exchange and transmission of facts, opinions, feelings, etc. (conscious as well as unconscious), including the development of an interpersonal relationship and mutual trust within the communication process.

Good and effective communication is essential for the provision of good medical care. The importance of communication for physicians in a sickness certification or disability assessment setting is possibly even more pronounced, as has been clearly illustrated by O'Brien et al. [4]. In their interview study, patients who visited a general practitioner for a sick note indicated that a good doctor-patient relationship was important to them, as were opportunities to talk about various illness-related issues. Moreover, many of these patients stated that doctors lack the necessary time and knowledge for this purpose [4]. On the other hand, doctors also experience difficulties with the relationship during sickness certification consultation, but they believe that communication is one of the most important aspects of sickness certification as well [5].

Very few studies have focussed on the importance of communication during assessment interviews or sickness certification consultations [6], but it has been found that the way in which doctors approach their patients (i.e. the degree of proactive communication: taking the initiative and anticipating the claimant) when discussing return to work was related to the duration of the workers' compensation benefit. More proactive communication was associated with a shorter period of disability benefit, albeit only in the first thirty days [7]. Moreover, the fact that communication is, indeed, important for both the social insurance physician and the claimant, was illustrated by the finding that many of the complaints made by claimants to the social insurance company concerned being treated discourteously by social insurance physicians or by labour experts [8]. Lippel investigated the possible beneficial and adverse effects of the sickness compensation assessment process for injured employees. These claimants mentioned mental health problems as the most pronounced adverse effects of the assessment process. Stigmatisation, prejudice and lack of support were all contributing factors [9]. Moreover, it has been suggested that increased transparency of the medical disability assessments can result in less complaints about malpractice, by increasing the claimant's satisfaction with and acceptance of the outcome of the assessment [10]. Greater transparency might also increase their general acceptance in political decision-making and society in general [11].

In studies focussing on social insurance physician-claimant communication, the intentions and behaviour of the claimants were found to be just as important as the intentions and behaviour of the physicians. For example, the 'Eurocommunication Studies' focussing on communication between general practitioners and patients in ten European countries, found that it was not primarily the health care system, but patient characteristics that have the greatest influence on communication. Conversely, the contribution of physician characteristics was found to be of less importance [12]. Other important characteristics are age, gender, and social class. Examples of physician-specific characteristics are medical speciality and income, and examples of patient-specific characteristics are prognosis, level of education and health beliefs [13].

### The behavioural model

To gain insight into communication behaviour during disability assessment interviews, a behavioural theory (a theory according to which behaviour is learned instead of being innate) was taken as a starting point. There are many common aspects of behavioural theories (also called motivational theories or cognitive theories; for example [14]). Well known theories, such as the Social Cognitive Theory (SCT) [15], the Theory of Reasoned Action (TRA) [16,17], the Theory of Planned Behaviour (TPB) [18], and the Attitude/Social influence/self-Efficacy model (ASE model) [19,20], for example, share the concepts of attitudes, behaviour, intentions with regard to behaviour, self-efficacy, social influence, skills, and barriers. Attitudes refer to beliefs or consistent, external evaluations of another person, action, or idea; intentions are the willingness to adopt a certain behaviour; self-efficacy is the confidence and ability to be able to act adequately in a given situation; social influence is the influence of social norms and beliefs of relevant others on a person's actions; skills concern the capacity to adopt certain behaviour; barriers are potential obstructions that could prevent the occurrence of certain behaviour. Of all the theories mentioned, the TPB and the ASE model are the most recent and comprehensive models. The TPB is based on three types of beliefs: (1) beliefs about and evaluations of the likely results of behaviour, which lead to positive and negative attitudes towards behaviour; (2) beliefs about and evaluations of norms and expectations of others, which lead to compliance with or rejection of these subjective norms; and (3) beliefs about behaviour-facilitating or behaviour-impeding factors and their strength, which lead to perceived behavioural control. The combination of attitudes, subjective norms, and perceived behavioural control (also referred to as self-efficacy) leads to behavioural intentions, which then lead to behaviour [17,18]. The main difference between the TPB and the ASE model is that the latter explicitly takes the influence of (objective) skills and barriers into account, whereas the TPB does not. However, the TPB has been studied more extensively.

The applicability of the TPB to communication behaviour in medical encounters has been assessed in several reviews, for example by Perkins et al. [21] and Eccles et al. [22] who investigated the relationship between intentions and behaviour. Physician-patient communication was investigated (i.e. education of the patient by the physician) in one study [23] in the Perkins et al. review [21], and it was concluded that the intentions of the general practitioners to provide patients with information were related to their attitudes and, in combination with self-efficacy, also to their behaviour. One study in the Eccles et al. review [22] concerned physician-patient communication in terms of patient education [24]. From the results of this study it was concluded that the TPB (e.g. self-efficacy regarding the education of patients) is a better predictor of intentions and future behaviour than the TRA.

Godin et al. [25] pointed out the weaknesses of both reviews [21,22] and they performed another review of many social behavioural theories. They identified six studies in which physician-patient communication was included, for instance by providing education and addressing mental health problems. It is remarkable that all of these studies used the TPB as their theoretical basis. The review [25] resulted in two important conclusions. Firstly, it showed that the efficacy of the TPB in predicting intentions and behaviour differed when different physicians participated in the study, different behaviour was studied, different methodology was applied, etc. Secondly, it nevertheless seems possible to predict the intentions and behaviour of health professionals on the basis of the social behavioural theories. The authors conclude that the TPB provides a good theoretical framework with which to predict behaviour [25]. In the field of sickness certification and social insurance medicine, we are not aware of any reviews that have been carried out to evaluate the application of the TPB to communication behaviour. We do, however, know of one study in which the TPB was applied to communication behaviour. Croon and Langius [26] studied the process of sickness certification assessment by social insurance physicians. They took the TPB as a starting point, because they wanted to find out why social insurance physicians assess in a certain way, and were therefore interested in their motivation. They found the TPB very useful [26].

The TPB has also been applied to assess patient behaviour by many researchers. It was used by Munro et al. [14] in their review of adherence to medication, and by Brawley and Culos-Reed [27] in their review of adherence and behaviour change. As will be explained below, the unique features of the contact between a social insurance physician and a claimant, compared to contact between other doctors (such as general practitioners or specialists) and their patients, support the choice of the TPB as a basis from which to investigate social insurance physician-claimant communication.

### Specific features of social insurance physician-claimant communication

The core concept of the present conceptualisation is communication behaviour, and in the social insurance physician-claimant contact there are two important aspects of this behaviour: "to gather sufficient information ... in a caring way" ([28], p. 1118). In other words, according to the Ong et al. review [29], the two main purposes of communication behaviour are "(a) creating a good inter-personal relationship and (b) exchanging information" (p. 903). From the social insurance physician's point of view, these two perspectives could be summarised respectively in the interview as patient-centred behaviour (i.e. behaviour that puts the patient and his/her concerns, perspective and information needs first), and physician-centred behaviour (i.e. behaviour that puts the physician's perspective and information needs first) [28]. The distinction between the two perspectives resembles the division in health care between instrumental (also referred to as task-oriented, paternalistic, or disease-oriented) and affective (also referred to as patient-oriented) patient-doctor relationships [e.g. [30-32]]. Instrumental relationships concern aspects of the relationship between the social insurance physician and the claimant that explicitly serve a goal (information-giving and information-seeking), and affective relationships concern collaborative, social-emotional aspects of the relationship between the social insurance physician and the claimant (positive and negative social talk). This also resembles differences in psychotherapeutic approaches, such as person-centred or client-centred psychotherapy and the more directive therapies. The instrumental model used to be a popular approach in medicine, but the affective approach is now more common [33,34]. However, different patients might prefer a different type of approach, depending for instance on the nature of their health complaints [35].

Although both the instrumental aspect and the affective aspect are important, the main focus of social insurance assessment interviews is an instrumental aim, i.e. gathering information to make the most accurate assessment of the functional capacity of the claimant, whereas in curative medicine there is often an equally strong focus on the affective aim, i.e. empathy, because patients often have a great need for reassurance. Within the assessment, the social insurance physician's main task is to assess the claimant's work capacity in relation to the medical disabilities, and not to cure or care for the claimant. Van den Brink-Muinen et al. [12,31] also concluded from their international comparison study that communication patterns between Dutch general practitioners and their patients are oriented towards instrumental behaviour (e.g. giving information and advice). Affective behaviour was also observed, but to a lesser degree than in other European countries [31]. Of course, the claimant might also ask for information, for example about the assessment process and the outcome (e.g. method of assessment, perceived work capacity, consequences for disability benefits, etc.). In addition, the claimant has an explicit or implicit need for a certain degree of empathy (e.g. someone to listen to his/her worries and frustrations, reassurance, emotional support in talking about disabilities), and possibly needs to be motivated or slowed down with regard to job performance. In this respect, the social insurance physician's background knowledge and experiences could, in general terms, be seen as his/her intentions during the communication, his/her self-efficacy, his/her skills, and perhaps even the social influence of others, such as colleagues and the employer.

Social insurance physicians generally work under substantial time-restrictions, and in some cases they only meet the claimant once, the latter unlike other physicians, such as general practitioners or specialists. Therefore, the social insurance physician's previous experience of communication with claimants and intentions, or general and claimant-specific preferences with regard to this communication, will have considerable influence on the communication behaviour during each specific contact. Moreover, the physician and the claimant have no choice with regard to participation in the assessment interview. They are thus dependent on each other, and whether or not they like each other initially - whatever the reason may be - will influence their communication. Empirical findings from social psychology research suggest that similarities in attitudes and behaviour are important in first-time encounters between people, and lead to better communication and personal attraction. This also applies to many other similarities in attitudes and behaviour [36-39], and can help to solve language problems and remove emotional barriers. It is important to note that these similarities not only increase the effectiveness of the exchange of information, but they also influence the emotional relationship: similarity in behaviour leads to personal attraction between people. Moreover, research findings indicate that this personal attraction is closely related to feelings of security and trust [40], and that during medical encounters, similarities between physicians and their patients enhance their communication and their satisfaction with it [41]. However, cultural differences cause problems in communication [42]. Similarities or differences between the social insurance physician and the claimant might therefore influence the course of their communication. Especially, during a once only or occasional contact, or when there is limited time to establish a relationship, the physician must quickly make the claimant feel at ease in order to obtain the information that is necessary for the assessment. In such situations the claimant has little time to gain trust in the physician in order to feel comfortable enough to talk in detail about his of her medical problems.

In social insurance medicine, not only the communication behaviour itself, but also satisfaction with that behaviour may play an important role, because to a certain extent satisfaction determines how, and how efficiently information is exchanged. If a physician is unhappy with the communication during an assessment interview, he is more likely to change his behaviour and look for different ways in which to gather the necessary information. Similarly, the satisfaction of a claimant will probably influence his or her willingness to provide the physician with the necessary information. Moreover, assessment interviews are daily routine for social insurance physicians, whereas they are only incidental for claimants.

From the perspective of the physician it is important to note that there are two distinct groups of claimants. Those in the first group have had previous experience of an assessment interview, which means that they already know what to expect (their expectations and attributes are perhaps more realistic), or at least know more about how an assessment interview is conducted (whether good or bad) and will behave accordingly. For this reason, they will probably feel that they have more control over the interview and the communication. Their intentions and preparations will probably differ from those of the claimants in the second group, for whom it is the first assessment interview for a disability benefit. For example, claimants with previous experience will probably base their expectations on visits to physicians in general or a description of the procedure, which may be based on positive or negative stories about assessment interviews.

### An overview of the conceptualisation

**Figure 1:**
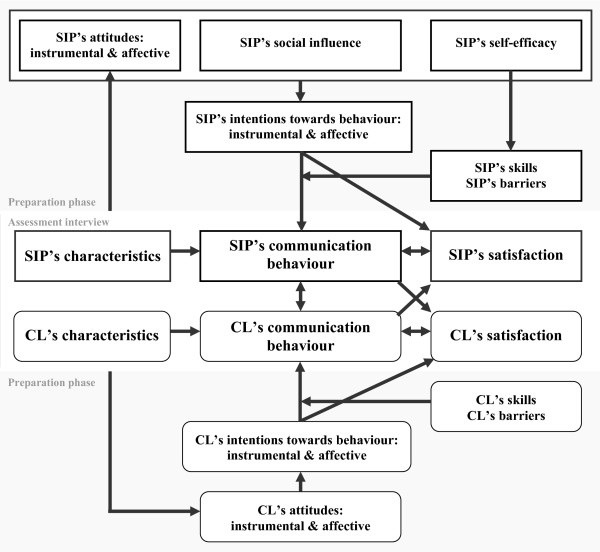
**Behavioural model regarding communication between social insurance physicians (SIP) and claimants (CL) during assessment interviews**.

In summary, it can be concluded from the three reviews discussed above [21,22,25] that the TPB is an appropriate starting point for investigating the key components of physician-claimant communication behaviour. The theoretical framework we therefore propose to use will be explained below, and is presented in Figure 1. In general terms, the model states that a combination of attitudes to communication behaviour, social influences, and self-efficacy, leads to the intentions of social insurance physicians to adopt that communication behaviour. Self-efficacy influences the skills to adopt the behaviour, and depending on these skills and on barriers preventing the physician from adopting it (the concepts of skills and barriers are derived from the ASE model), these intentions will or will not lead to several core aspects of actual communication behaviour. The specific characteristics of social insurance physician-claimant communication support the use of this general theoretical framework. As the figure shows, we make a distinction between the assessment interview itself and the preparatory phase, in which the physician and the claimant mentally prepare for the assessment interview independently. The preparatory phase for the physicians consists of their attitudes and intentions with regard to communication with claimants in general. Both the instrumental, physician-centred orientation and the affective, patient-centred orientation are included in those core-aspects. Furthermore, the physician will be influenced by other people, have a certain degree of self-efficacy, master specific skills, and experience specific barriers.

At the centre of the model is the actual assessment interview, during which both the physician and the claimant are present. This is the action phase that follows the preparatory phase. The core issue of an assessment interview is the communication behaviour, and how this is perceived and evaluated by the people involved. Since both people are present during the assessment interview and the exchange of information is a continuous, dynamic process, the model states that the behaviour of the physician influences that of the claimant, and vice versa. The psychological mechanisms of "transference" (the claimant expresses feelings, wishes and experiences towards the physician that are actually felt towards other people who are of were important in the claimant's life) and "counter-transference" (reactions from the physician to the claimant) might be involved here. Moreover, there will always be interaction between the occurrence of and satisfaction with the communication behaviour, both of which are constantly changing and influencing each other. This is in line with findings that the general consultation characteristics of patients and physicians might influence their satisfaction [e.g. [43,44]], and that satisfaction is related to a patient's perceptions of an encounter, but not to more objective observations [45]. Therefore, the core of our framework stresses the more subjective, perceived communication behaviour and people's evaluations of that behaviour (i.e. satisfaction), instead of objective, observable behaviour. The full theoretical framework that results is substantial, in that it covers the communication process as a whole, including the relationships between the different aspects and persons involved, and 'environmental' aspects, such as the personal characteristics of the people involved. This 'ecological approach' (i.e. an approach that states that behaviour results from multiple sources which interact, including the person himself/herself, other people, and the context, including the situation and environment), is advocated by Street et al. [46], who argue that an ecological approach is the most suitable method for describing physician-patient communication. They stress that from an ecological viewpoint all relevant influences on the communication are taken into account within the context of the medical consultation.

## The conceptualisation for social insurance physicians

In the following, we will conceptualise the theoretical model applied to communication behaviour during assessment interviews. We will do this for the social insurance physician and the claimant separately. A summary is presented in Table 1.

**Table 1:** Conceptualisation of the behavioural model regarding communication between social insurance physicians (SIP) and claimants (CL) during assessment interviews

	**Concepts**	**Conceptualisation to communication**	
		**Social insurance physician**	**Claimant**

	Intentions	Problem-solving communication style^1^ Insurance-technological communication style^1^ Careful communication style^1^ Intention to give in to CL^5^ Intention to force the own will on CL^5^ Intention to solve problems jointly with CL^5^	Problem-focussed, strategic coping^10^ Psychological distancing and avoiding^10^ Seeking social support^10^ Seeking practical support
	
	Attitudes	Practice-directed attitude^1^ Result-directed attitude^1 ^(sharing attitude^2^) Patient centeredness^2^ Distribution of responsibility^3^ Attitude towards own profession^4^	Relationship-focussed attitude^1^ Result-directed/information-focussed attitude^1^ Attitude regarding patient-centeredness^2^ Passive coping attitude^6^ Wait-and-see coping attitude^6^ Active problem-focussed coping attitude^6^ Attitude about expression of emotions^6^
	
Preparation before assessment interview(s) (preparation phase)	Social influence	Public opinion^16^ Opinion of colleagues^16^ Opinion of other SIPs Opinion of employing institute	Influence of other people^16^
	
	Self-efficacy	Self-efficacy trait about communication^8^	Self-efficacy^8^
	
	Skills	Skills related to disease/disability Degree of control over communication (process) Handling communication problems (perceptual)	Providing information^11^ Verifying information^11^ Presence of social support
	
	Barriers/support (in preparation)	CL's characteristics and skills Lack of information	SIP's characteristics CL's own characteristics (including disability)

	Barriers/support (in interview)	CL's characteristics and skills Other people who are present Lack of information (e.g. missing files)	SIP's characteristics CL's own characteristics (including disability)
	
	Behaviour	Instrumental communication behaviour Affective communication behaviour	Instrumental communication behaviour Affective communication behaviour
	
During assessment interview (action phase)	Satisfaction (appraisal of behaviour)	Focus on instrumental aspect (information exchange and making decisions): • Listening^15^ • Correctness^15^ • Clarity^15^ • Satisfaction with provided information^1^ Focus on affective aspect: • Empathy^15^ • Carefulness^15^ • Take CL seriously^12^ • Helping alliance^14^ • Trust and confidentiality^12^ • Knowledge-based trust^3^ • Identification-based trust^3^ • Satisfaction with cooperation^1^ General overall degree of satisfaction^13^	Focus on instrumental aspect (information exchange): • Listening^15^ • Correctness^15^ • Clarity^15^ • Satisfaction with provided information^1^ Focus on affective aspect: • Empathy^15^ • Carefulness^15^ • Being taken seriously as a CL^12^ • Helping attitude^14^ • Trust and confidentiality^12^ • Knowledge-based trust^3^ • Identification-based trust^3^ • Satisfaction with cooperation^1^ General overall degree of satisfaction^13^
	
	Personal characteristics	Age Gender Socio-cultural background Legal context	Number of previous assessment interviews Age Gender Socio-cultural background Level of education Personality characteristics^9^

^1 ^[26]; ^2 ^[32,88]; ^3 ^[54]; ^4 ^[93]; ^5 ^[56]; ^6 ^[89,90]; ^8 ^[78]; ^9 ^[94]; ^10^[82]; ^11^[85]; ^12^[49,50]; ^13^[51]; ^14^[52]; ^15 ^[48]; ^16 ^[95]

### Behaviour

The core concept of the present conceptualisation, based on the TPB, is communication behaviour, which occurs when the social insurance physician and the claimant meet during the assessment interview. At this point, the communication process takes place, and both people will have an opinion about the content and process of this communication behaviour. Given the afore mentioned arguments, both the instrumental and the affective dimensions of communication behaviour are important. Instrumental behaviour, for example, includes applying technical skills such as the specific method of asking questions and summarising the information the claimant provides. Examples of affective behaviour are expressing empathy and making contact in a respectful way. Derived from the TPB, intentions with regard to the communication (i.e. assessment styles), and the physician's communication skills and perceived barriers are conceptualised to influence the communication behaviour. Assessment styles, and especially the preferred assessment style(s) of the physician are believed to influence his perception of the claimant's communication behaviour and thus his/her appraisal thereof. The same applies to barriers, such as expectations based on knowledge of the claimants records or previous experiences of similar claimants. Personal intentions might 'precondition' perception of the other people's intentions, and hence their behaviour. This is in line with the results of Adler's overview [47], in which he found that empathy was the result of mutual responses. We postulate that the communication behaviour of the claimant will influence that of the physician and vise versa (which we will explain below). The physician will probably change his or her own behaviour, either consciously or unconsciously, in reaction to the behaviour of the claimant [47]. For instance, if the physician dislikes the claimant's behaviour, he or she will attempt to change it or minimize the negative consequences. Moreover, the physician's satisfaction with the communication is also influenced by the claimant's behaviour.

Summarizing, the physician's communication behaviour influences and is influenced by the claimant's communication behaviour. In turn, the claimant's behaviour influences the physician's satisfaction with the communication, which will subsequently influence the behaviour of the physician, at which point the circle is closed.

### Satisfaction

Satisfaction includes the evaluation of the consequences that are directly associated with the performance of the behaviour. The degree of physician's (dis)satisfaction with the communication with the claimant will depend on a combination of two factors. Firstly, it depends on the perception and appraisal of the claimant's behaviour, and secondly, on intentions, or more specifically, the degree to which these match the claimant's behaviour.

Social insurance medicine practices are a good starting point for the different domains of satisfaction. For instance, in the Netherlands a periodical monitoring survey that is carried out by the research centre of the Institute of Employee Benefit Schemes was developed especially for use in this context, and optimisation is still in progress. It includes six behavioural aspects of satisfaction with the communication during assessment interviews: listening, empathy, correctness, clarity, carefulness, and expertise [48]. Because the dimension 'expertise' partly overlaps with other dimensions (e.g. asking appropriate questions is one aspect of this dimension and information exchange is also an aspect), 'expertise' is not included in our conceptualisation.

Verbeek et al. [49] added the aspects of "being taken seriously" and "trust and confidentiality", based on their review of the literature on consumer satisfaction with occupational health care. Moreover, they conclude that satisfaction is a multidimensional construct, and they therefore recommend that specific dimensions of satisfaction as well as general dimensions of satisfaction are taken into account [49-51]. In primary health care, Van der Feltz-Cornelis et al. [52] stressed the importance of effective and helpful communication in the physician-patient relationship. They followed the psychotherapeutic concept of the Helping Alliance, i.e. considering the psychotherapeutic relationship as a means by which a health professional can engage with the patient, and suggest that satisfaction with the helping attitude of physicians is an important aspect of patient satisfaction in primary care [52]. As we have already pointed out, trust is important in the social insurance physician-claimant communication. Nauta [53,54] made a distinction between knowledge-based and identification-based trust. Knowledge-based trust is trust in the competence of the other person, and identification-based trust is trust in the way the other person communicates, in other words affect-based trust [53,54]. Both types of trust are likely to be present in social insurance physician-claimant communication.

All the above mentioned components of satisfaction can be considered as part of the instrumental dimension of satisfaction or part of the affective dimension of satisfaction. Croon and Langius [26] demonstrated that these two dimensions are also explicitly perceived by claimants, who distinguish (1) a dimension focussing on the actual provision of information to them during the communication; and (2) a dimension focussing on the inter-personal communication and negotiation during the assessment interview.

Summarizing, the appraisal of communication behaviour is believed to be a multidimensional concept. Several aspects could be distinguished regarding: (1) the exchange of information and decision-making (instrumental dimension), and (2) the inter-personal relationship (affective dimension). For the first aspect, listening, correctness, and clarity are relevant domains of satisfaction, as is satisfaction with the actual provision of information. For the second aspect, empathy, carefulness, being taken seriously, helping alliance, general trust and confidentiality, knowledge-based trust, identification-based trust, and satisfaction with co-operation in the communication are believed to be important concepts. Furthermore, overall satisfaction should be taken into account.

### Intentions, skills, barriers

According to the TPB, behaviour is influenced by intentions to adopt that behaviour, and this relationship is mediated by skills and barriers. Social insurance physicians will have habitual and standard methods for exchanging information with claimants, since this represents a substantial part of their job. Intentions with regard to communication behaviour are therefore conceptualised as habitual communication styles during the assessment interviews, or in other words as specialised assessment styles. This is in agreement with the conceptualisation according to Croon and Langius [26], who proposed that the general behavioural intentions of social insurance physicians could be made explicit as their assessment styles. They defined 18 assessment styles with four underlying dimensions. The most professional style is the problem-solving style, which is defined as a preference for effective problem-solving, together with the claimant. It includes providing information and paying attention to the content of the assessment interview. The three other dimensions they proposed are of a more bureaucratic nature. The dimension of carefulness in handling the claimant consists of giving information about the course of the assessment interview, about the assessment itself, and about relevant laws. The insurance-technological dimension encompasses social, insurance-technical and workload/work capacity aspects, implying that both the instrumental and the affective aspect of the intention with regard to communication are represented. The knowledge-handling dimension concerns knowledge about disability benefit laws, medical disciplines, and occupational health disciplines. However, this dimension is not relevant, because this knowledge is not needed for communication during the assessment interviews, and is more applicable to the assessment procedure as a whole [26,31].

In the context of the assessment a lot is at stake for the claimant, and the opinions of the physician do not necessarily match those of the claimant, so it is not unlikely that differences of opinion might occur. It is clear that the way in which the physician handles small (and serious) conflicts during an assessment interview will influence the well-being of both parties [55,56]. For instance, a relationship between communication problems and (dis)satisfaction has been found in general health care [57]. The way conflicts are dealt with may influence the claimant's trust in the physician, especially in such a 'critical situation' as an assessment interview [58-60]. These findings are in line with the opinions of De Dreu et al. [56], who found that the style of handling conflicts is reflected in a combination of the degree of concern for yourself and that for others. These combinations include giving in to the claimant (high concern for the other and low for oneself), forcing the own will on the other person (high concern for oneself and low for others), and trying to solve the problem together with the claimant (high concern of self and others) [56]. Each social insurance physician will have his or her own preferences or intentions dealing with conflicts.

Since skills and barriers play a similar role - they are in a way the two sides of the same coin - they are linked together in the model. However, skills and barriers do differ in their conceptualisation. The importance of skills in the communication is emphasised by the many training courses in communication skills for physicians that have been developed and tested [e.g. [61-64]]. It is clear that the physician's skills with regard to the claimant's disease or disability might influence the communication [65]. Moreover, the degree of control the physician has in general over the communication during an assessment interview, as well as the physician's ability to change direction and handle problems during the interview are relevant skills. This agrees with the general distinction made by Kurtz (2002) of three types of skills: content skills, process skills, and perceptual skills. Content skills refer to the physician's basic medical knowledge, including the content of the questions asked, the information that is given, and the answers that are received. Process skills concern the way in which questions are asked, how to explain things, how to listen, and how to build up a relationship with the claimant. Perceptual skills concern the content and awareness of the physician's own thoughts and feelings.

Barriers previously experienced by physicians or barriers they have trouble dealing with, could be the result of other people being present during an assessment interview, for instance a claimant's relative or partner, or a union member, who might hinder the interview, for example because of unwanted participation [e.g. [66]]. Other barriers created by the claimant might be level of education, language restraints, family members functioning as an interpreter, and the diagnosis from curative health care. The expectations and experiences of the claimant are also important; for instance, previous experiences of visits to social insurance physicians (good or bad), and media reports about social insurance medicine [e.g. [67]]. Swartling, for example, reported that the societal attitude to sickness certification and benefits is an important barrier for sick-listing, according to Swedish general practitioners [6].

On the one hand, such barriers occur frequently, and could have a negative influence on obtaining information from the claimant or on the atmosphere during the interview [68]. On the other hand, some aspects might be supportive, instead of forming a barrier. Examples of this are that other people who are present help to explain things to the claimant and clarify the information the claimant gives (e.g. family member, trainee, or colleague), or claimants with a high level of education.

### Attitudes, social influence and self-efficacy

According to the proposed theoretical framework, the physician's intentions to exchange information in a certain, habitual way (i.e. assessment styles) are derived from a combination of three components: (1) attitude to the communication during the assessment; (2) social influences; and (3) self-efficacy, which influences the assessment style of social insurance physicians as well as the skills and barriers they encounter.

As was explained above, Croon and Langius [26] used the TRA and the TPB as a basis to study the relationship between the attitudes and behavioural intentions of social insurance physicians. The content of their practice-directed attitude and result-directed attitude is directly related to the communication. A practice-directed attitude defines the physician's aim to avoid conflict and to negotiate with claimants, taking the disability as a starting point for the assessment. The result-directed attitude is pragmatic, and aimed at helping the claimant to find a solution to the problems (e.g. better working conditions, assistance with return to work).

Furthermore, an "attitudinal component of patient-centeredness" [69] is believed to exist. More tangible, an instrumental and an affective dimension can be distinguished in the physician's attitude [69]. This is in agreement with the opinions of Krupat et al. [32], who studied attitudes in doctor-patient relationships and made a distinction between patient-centeredness and disease-centeredness, or in their own words, between a "caring" and a "sharing" element in the doctor-patient relationship [26,32].

The social insurance physician's task is to evaluate the degree of the claimant's disability, which has important implications for the claimant, and makes the relationship unequal by definition, as opposed to the purpose of a medical consultation. Equality in the communication might be conceptualised according to Nauta [54]. One of the recommendations she makes in her study focussing on co-operation between occupational physicians and general practitioners is to maintain a clear distribution of responsibility. Applied to equality in the physician-claimant contact, the question that arises would be whether the responsibility for an effective communication lies with the physician or (also) with the claimant. This distribution of responsibility is an important aspect, because of the shift in general health care from a paternalistic view of the patient to a more patient-oriented view [33]. Although the social insurance physician's attitude towards his or her own profession [70] is not directly related to communication, it may play a central role in the assessment interview. Nauta, for example, found that identification with one's own profession results in greater feelings of responsibility [54]. Research results confirm this concept by demonstrating that job perception and job satisfaction influence doctor-patient communication [71]. For instance, Grol et al. [72] found that general practitioners with a positive attitude towards their job were more open and paid more attention to the psychosocial aspects of care, whereas those with a negative attitude gave less explanation to their patients. Job satisfaction may also influence patient satisfaction with the care that is provided as found by Haas et al. [73] in a study population of general internists.

In addition to attitudes, social influences are also believed to determine assessment styles or intentions with respect to communication behaviour during an assessment interview. Based on research findings, it would be expected that social influences co-determine how the physician performs his/her job. For instance, the medical professions are criticised regularly, public mistrust exists [e.g. [74]], and physicians feel a lack of support from society, politicians, the media, etc. [75,76]. Moreover, patients are active health care utilisers, health information is easily accessible to them and they have high expectations [75]. A combination of these three aspects will probably influence the way in which the physician communicates with claimants [71]. More specifically, public opinion, the opinion of colleagues, and the policies, standards, and values of the company for which the physician works could be important sources of social influence [77]. This social influence could affect three aspects of the assessment interview: (1) the skills of the physician compared to those of others; (2) his/her knowledge; and (3) his/her experience.

The last factor that influences the physician's assessment styles is self-efficacy. According to Bandura, self-efficacy is domain-specific, and should thus be conceptualised. Therefore, in line with Scholtz et al. [78], we define self-efficacy as a global and stable confidence in the ability to cope with the communication with claimants during assessment interviews. Self-efficacy is regarded as a one-dimensional global construct [78], and is thus conceptualised as a type of trait, resulting from previous positive and negative experiences in communication with claimants [79].

### Personal characteristics

The personal characteristics of the social insurance physician are not incorporated in the TPB theory. They are conceptualised to exert their influence on the 'communication circle' that originates during the disability assessment interview.

The most important and pronounced personal features which can be similar are age, gender, and socio-cultural background. Research supports the assumption that these personal characteristics are relevant with regard to similarity in the communication between physicians and their patients [41,46]. Furthermore, the legal context in which the assessment interviews take place could be considered a feature that also corresponds with the characteristics of the social insurance physician.

## The conceptualisation for claimants

Because not every aspect is visible for the social insurance physician, the conceptualisation for claimants will be only partly analogous to that for the social insurance physician, and only part of the TPB will be conceptualised for the claimant. Attitudes and intentions are the core concepts of the TPB, so it is likely that the physician will be aware of the influence of the claimant's attitudes and intentions during the assessment interview. The other aspects of the model will have their influence through the intentions. The only exceptions are the skills and barriers, which influence the relationship between intentions and behaviour. Because of the direct influence of skills and barriers on behaviour, these are included in the claimant's side of our theoretical framework. The included aspects are intentions with regard to behaviour, attitudes, skills, and barriers. The way in which claimants cope with assessment interviews - their communication behaviour and their satisfaction with the communication - is also included, because this is directly relevant, visible, and experienced by physicians. The application of these aspects of the theoretical framework to claimants will now be presented.

### Behaviour and satisfaction

Because it is believed that the dimensions of patient or claimant satisfaction are mostly similar to the dimensions of physician satisfaction [49], the communication behaviour and perceived behaviour of the claimant during the assessment interview is conceptualised in the same way as that of the physician. Communication behaviour and satisfaction with that behaviour are conceptualised as multidimensional, with the same dimensions as for the physician.

### Intentions, skills and barriers

Although attending an assessment interview is not a routine activity for the claimant - as it is for the physician - the claimant's normal way of communicating will probably be similar to the way in which he or she will communicate with the physician. Moreover, we know from research that the communication style of the patient is equally important as that of the physician [46], and that this communication style (i.e. intention) is also the claimant's way of handling communication in general and communication with other physicians in particular. Folkman and Lazarus [80] argue that before stressful encounters - such as examinations during a study, and also assessment interviews - people tend to handle the situation in an instrumental way (problem-focussed), and afterwards they tend to display more emotion-focussed coping (e.g. seeking social support). This conceptualisation is supported by Carver et al. [81], who discriminate between the use of instrumental support and the use of emotional support (among other types of coping), and by the results of studies in general health care, as mentioned above. Thus, the claimant's intentions with regard to communication can be both instrumental and affective. Bramsen et al. [82] made a more detailed distinction: problem-focussed coping according to a preceding plan, psychological distancing and avoiding (i.e. mentally creating distance between oneself and the environment), and seeking social support [82]. These last two styles are forms of emotion-focussed coping, which Miller [83] referred to as a "blunting" and a "monitoring" coping style, respectively. Patients who blunt will avoid information, and those who monitor are very alert and are keen to receive information. According to Nordin et al. [84], these coping styles moderate satisfaction with the communication behaviour of medical staff. We therefore suggest that claimants, apart from seeking social support, may also intend to seek practical support. For example, they may intend to gather information about the assessment interview before attending, they may ask someone to go with them to the assessment interview, or they may practice beforehand by giving the relevant information to someone else.

The skills and barriers that claimants experience are conceptualised to affect the relationship between intentions with regard to behaviour and actual behaviour, and as we mentioned earlier, some connections do exists between these two factors, but they also have their own specific characteristics. Cegala et al. [85] found that training patients' skills in handling medical interviews resulted in a more patient-controlled communication, and that trained patients gave physicians more detailed information about their disabilities and were more able to summarise the information they received. Thus, training the skills needed to seek, provide, and verify information, seems to be important [85]. For claimants, seeking information is not usually the primary goal during an assessment interview, so this skill is not included in the framework, whereas the other two are. Some examples of such skills are command of language, ability to explain their functioning, and ability to understand the physician. As the CanMed Physician Competency Framework states, it is important that physicians can gather information and understand it, as well as establish a good relationship with the patient [86]. Presumably, the same applies to claimants, since their claim depends on the physician's assessment. Being able to influence the course of the interview and to handle difficult situations (solving problems) seem to be particularly relevant skills for claimants [86,87].

Claimants might anticipate several barriers which may be related to the characteristics of the physician, for instance a different socio-cultural background from that of the claimant or the use of difficult language. The claimant's own characteristics, possibly related to the disability, could also form a barrier, such as concentration problems or physical fatigue.

### Attitudes

Parallel to the importance of the physician's attitude towards communication, the attitude of the claimant might also influence the communication during an assessment interview. Claimants might have different attitudes with regard to the role of the physician in the communication, and these might hinder or aid the physician. These attitudes can be conceptualised analogously to the attitudes that the social insurance physician has about his or her own role in the communication. Therefore, the attitude of claimants towards the communication is conceptualised as relationship-focussed, result-directed/information-focussed [26], and focussed on the patient-centeredness of the physician [88]. Relevant aspects of such attitudes are: expectations about support, listening, and asking questions for the relationship-focussed attitude; asking and thinking about return to work and talking about possibilities of return to work for the result-directed attitude; and expectations about reassurance and a good atmosphere for the caring attitude. As mentioned above, claimants who have attended an assessment interview before and those who have not will probably have different attitudes.

In addition to the attitude towards the contribution of the physician to the communication, the claimant will also have an attitude towards his own contribution to the communication. We refer to this as the coping attitude, because it concerns the way in which the claimant anticipates handling (coping with) the communication. Moreover, claimants will use certain general coping strategies while preparing for the assessment interview. Kloens [89] advised psychologists to take general coping strategies into account during the assessment of a patient. He distinguished three components of coping attitudes: a passive avoiding coping pattern of responding to the assessment, a problem-focussed coping pattern, and an emotion-focussed coping pattern, which includes the degree of seeking social support and expressing emotions. The passive avoidance coping attitude could then be sub-divided into a passive coping attitude and an avoidance (wait-and-see) coping attitude, in line with the Schreurs definition [90].

### Personal characteristics

We have already stated that the number of previous assessment interviews a claimant has experienced, is an important claimant characteristic, explaining the difference between a first-time claimant and a claimant who has already attended one or more interviews. As in the conceptualisation for social insurance physicians, prominent characteristics which may be similar for claimants and social insurance physicians are age, gender, and socio-cultural background [41,46]. Moreover, the claimant's level of education might influence the communication, for example because claimants with a higher level of education are generally more assertive, and physicians tend to give them more information [31,41,46]. In addition to attitudes and intentions, the claimant's personal characteristics will influence the communication. For example, an anxious claimant is likely to communicate quite differently with the physician than a depressed or confused claimant [31]. This depends on the claimant's 'locus of control' (i.e. a personality trait indicating the degree to which gains are thought to result from one's own efforts or considered to be random events; according to the claimant, for example, who is responsible for whether or not the claimant will receive a disability benefit), and the related degree of control experienced in the communication.

## Discussion

We have presented a theoretically conceptualised model, based on the TPB, to study the communication behaviour of social insurance physicians and their claimants during (the preparation of) medical disability assessment interviews. This model will help us to understand the communication process during assessment interviews, and how this communication could go wrong, and we have made suggestions that could be appropriate to improve this communication. Because the conceptualisation specifically focuses on non-curative medicine, with social insurance medicine as an example (a field in which to our knowledge no such conceptualisation has been applied), this model might be of assistance in future research in this context.

### Strengths and weaknesses of the behavioural model

Our choice to make use of existing behavioural theories, particularly the TPB, has advantages as well as disadvantages, both of which have been stressed by several authors. For instance, Ogden [91,92] argued that behavioural models are pragmatic in guiding research because, although they are considered to be an appropriate basis for the development of interventions to change certain types of behaviour, their conceptual basis is less sound. However, Ogden's arguments based on problems in applying these theories and measuring the concepts, were refuted by Ajzen and Fishbein [92]. The only argument they did not refute is that the concepts are not specific enough. We believe that we have countered this argument by specifying the concepts adequately in our proposed model. Moreover, in our opinions, the advantages of using the TPB to understand the communication processes in social insurance medicine (e.g. focus on the instrumental as well as the affective dimension, application in studies in related areas, and the amount of detail that is possible within the model) far outweigh the disadvantages. This is mainly because the conceptualised model is pragmatic in guiding further research, functional in formulating hypotheses, and useful in developing interventions to improve social insurance physician-claimant communication.

The resulting theoretical framework is quite comprehensive. In order to ensure that the model was feasible, we chose not to assume relationships between the conceptualisations of the aspects within the framework (e.g. the relationship between a problem-solving communication style or an insurance-technological communication style, and a practice-directed attitude or a result-directed attitude). The comprehensiveness of the framework may be both positive and negative. The positive aspect of a comprehensive framework is that there is a choice of focus, i.e. our conceptualisation is suitable for different types of research. For instance, the focus could be on the social-emotional or on the task-oriented aspects. Moreover, parts of the framework could be used for more in-depth evaluations, for instance in observational or qualitative studies. With regard to the negative side, when research is based on such a comprehensive model, there is a danger of wanting to investigate too much all at once. This means that studying the model as a whole implies a more general, less in-depth, procedure with, for example, questionnaires or structured interviews.

### Implications for future research

Based on this conceptualisation, we hypothesise that the main relationships, indicated by arrows in Figure 1, will be found in an empirical test of the conceptualisation. According to the TPB theory, it is expected that the communication behaviour of social insurance physicians during assessment interviews can be predicted from a combination of their attitudes, experienced social influence, self-efficacy, intentions with regard to behaviour, skills, and barriers in the communication with claimants in general. Analogously, it is expected that the communication behaviour of claimants during the assessment interview can be predicted from their attitudes, intentions, skills, and barriers in the communication with social insurance physicians, or in the communication with physicians in general if they have had no previous experience with social insurance physicians. During the assessment interview, it is hypothesised - according to the proposed conceptualisation - that the communication behaviour of both the social insurance physician and the claimant will be the result of their input during the preparatory phase, their personal characteristics, and the degree to which these match those of the other person, their satisfaction with the communication behaviour, and the other person's behaviour.

When the relationships in the conceptualisation have been tested empirically, the TPB-based model for communication behaviour in social insurance medicine can be applied in empirical studies to obtain more insight into communication behaviour in non-curative medicine. We also expect that the concepts and relationships in the conceptualised model could be used in a communication skills training course for social insurance physicians. The model may help these physicians to recognise communication behaviour, and to intentionally and purposefully adapt their communication behaviour to their task when assessing the functional capacity and medical disabilities of claimants.

## Conclusion

We have presented a conceptualisation of a behavioural model, derived from the TPB, for social insurance physician-claimant communication. This conceptualisation was based on studies focussing on physician-patient communication and the specific characteristics of social insurance physician-claimant contacts. Of course, just like any model, this model is merely a simplified representation of the reality. Although, obviously not every aspect, dimension, or variation is represented in the framework, it provides ample insight to professional communication from the perspective of non-curative and social insurance medicine.

## Competing interests

The authors have no conflicts of interest that are directly relevant to the content of this article.

## Authors' contributions

HJvR wrote the manuscript. AJMS, JRA and AJvdB revised and commented on the manuscript. All authors have read and approved the final version of the manuscript.

## Pre-publication history

The pre-publication history for this paper can be accessed here:


